# Concepts, protocol, variations and current trends in surgery first orthognathic approach: a literature review

**DOI:** 10.1590/2177-6709.23.3.36.e1-6.onl

**Published:** 2018

**Authors:** Hafiz Taha Mahmood, Maheen Ahmed, Mubassar Fida, Adeel Tahir Kamal, Farheen Fatima

**Affiliations:** 1 The Aga Khan University Hospital, Department of Surgery, Section of Dentistry, Orthodontics Residency Program (Karachi, Pakistan).

**Keywords:** Orthognathic surgery, Le Fort osteotomy, Sagittal split ramus osteotomy, Mandibular osteotomy, Maxillary osteotomy

## Abstract

In the current era of expedited orthodontics, among many clinicians, tertiary care hospitals and patients, surgery first orthognathic approach (SFOA) has gained popularity. The advantages of SFOA (face first approach) are the reduced overall treatment duration and the early improvement in facial esthetics. In SFOA, the absence of a presurgical phase allows surgery to be performed first, followed by comprehensive orthodontic treatment to achieve the desired occlusion. The basic concepts of surgery early, surgery last, SFOA and Sendai SFOA technique along with its variations are reviewed in the present article. The recent advancement in SFOA in the context of preoperative preparation, surgical procedures and post-surgical orthodontics with pertinent literature survey are also discussed.

## RESUMO

Na presente era da Ortodontia de resultados acelerados, a cirurgia ortognática com benefício antecipado (COBA) ganhou popularidade entre muitos clínicos, hospitais terciários e pacientes. A vantagem da COBA é a redução da duração total do tratamento, juntamente com a melhora precoce da estética facial. Na COBA, a ausência de uma fase pré-cirúrgica permite que a cirurgia seja realizada antes e, só então, venha o tratamento ortodôntico abrangente para se alcançar a oclusão desejada. Os conceitos básicos de cirurgia primeiro, cirurgia por último, COBA e a técnica COBA de Sendai, bem como suas variações, são aqui revistos. Também são discutidos no presente artigo, junto com a revisão da literatura pertinente, os recentes avanços da COBA no contexto do preparo pré-cirúrgico, dos procedimentos cirúrgicos e da Ortodontia pós-cirúrgica.

## INTRODUCTION

Orthognathic surgery is the treatment of choice for correction of various dentofacial deformities. Conventional orthognathic surgery (COS) requires certain duration of presurgical orthodontics to alleviate the dental crowding, level the curve of Spee, decompensate the dental inclinations, remove any occlusal interferences and coordinate the upper and lower arches.^1^ ^,^ ^2^ Luther et al^3^ have reported an average duration of 17 months for presurgical orthodontics, while Dowling et al^4^ and O’Brien et al^5^ have found the mean duration to be 15.4 months and 25 months, respectively.

In addition to prolonged treatment duration, other disadvantages of presurgical orthodontics include gingival recession, gingival hyperplasia, dental caries, root resorption, deterioration in occlusal function, masticatory and speech discomfort and subsequent psychological problems due to delay in resolution of patients’ chief complaint.^6^ ^,^ ^7^ Moreover, there is a further deterioration in the patients’ facial profile during the presurgical phase which leads to a negative impact on the quality of life.^8^ The COS requires that comprehensive orthodontic treatment be carried out post-surgically for final detailing and settling of the occlusion, which leads to an increased overall treatment duration.^9^

The alternatives to COS include surgery early, surgery last and surgery first orthognathic approach (SFOA). Hernandez-Alfaro and Guijarro-Martınez^10^ described ‘surgery early’ as the technique that is indicated in subjects with severe dental crowding or complex three dimensional (3D) dental compensations caused by facial asymmetry, including dental midline deviations. The surgery is performed once crowding and transverse compensations are corrected with a minimal duration of presurgical orthodontics. The concept ‘surgery last’ approach is the modality indicated in patients who had previous comprehensive orthodontic treatment but are unhappy with their results and have decided to undergo surgery.^10^

**Figure 1:**
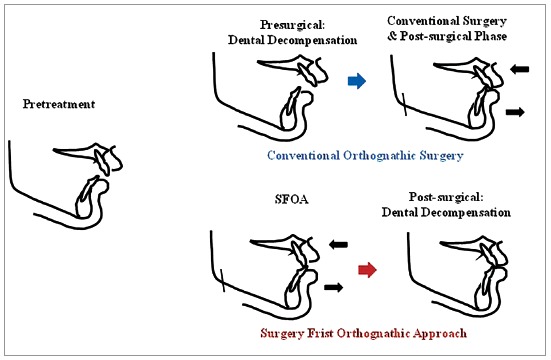
Various approaches for the treatment of skeletal deformity.

In SFOA, there is no presurgical phase; surgery is performed first followed by comprehensive orthodontic treatment to achieve the desired occlusion (Fig 1). This approach, also termed as the ‘face-first approach’, results in early improvement of the facial appearance.^8^ This leads to increased patient cooperation in the post-surgical phase.^11^ Additionally, with the absence of a presurgical phase, the patient has the opportunity to decide SFOA at their convenience. 

Recently among many clinicians, tertiary care hospitals and patients, SFOA has gained popularity due to no presurgical phase and reduced overall treatment duration. Peiro-Guijarro et al,^11^ in their systematic review, have reported a mean total treatment duration of 14.2 months with a range of 10.2-19.4 months for SFOA. With regard to complications, SFOA and COS are both comparable.^12^ However, Pelo et al^13^ have speculated that due to increased segmental osteotomies, the risk of complications with SFOA is slightly greater as compared to COS. 

Justus et al^14^ have reported increased blood flow levels during the healing process after surgery. This would result in increased bone turnover, a process similar to regional acceleratory phenomenon, that enhances the post-surgical orthodontic tooth movement. Moreover, Behrman and Behrman^15^ have presented the concept that when the underlying jaw deformities are corrected with SFOA, the normalized surrounding soft tissues accelerate the orthodontic tooth movement, which is also a factor responsible for decreased overall treatment duration. Nagasaka et al^16^ were among the first to actually carry out SFOA using miniplates for post-surgical orthodontic treatment. The increased range of orthodontic tooth movement in all dimensions is helpful in even correction of relapse that may have occurred post-surgically. 

The SFOA is indicated in highly motivated patients with minimal arch length discrepancy, mild to moderate transverse, vertical and sagittal discrepancies, with normal incisor inclinations and minimal dental compensations, to avoid interferences during the surgical correction.^11^ However, it should not be considered for patients with cleft related deformities, patients with high probability of development of CR-CO discrepancy and unilateral or bilateral cross-bite or scissor bite post-surgery.^12^

### SFOA protocol

SFOA requires efficient treatment planning, skilled model surgery and meticulous post-surgical orthodontics.^12^ It can be approached via two methods. The ‘surgical-driven’ approach corrects both the jaw and dental deformities via the surgical procedure.^17^ The ‘orthodontic-driven’ approach corrects the jaw deformity by surgery and the dental deformity via orthodontics.^16^ ^,^ ^18^ The initial protocol was recommended in 2003 at Tohoku University in Sendai city of Japan.^19^ It is an orthodontic-driven procedure that utilizes the miniplates in the form of skeletal anchorage system (SAS) for orthodontic movement following correction of the jaw deformity. The Sendai technique for SFOA may be summarized into preoperative, surgical and post-surgical procedures as follows: 

**a. Preoperative:**

» Diagnosis: The appropriate treatment goals for an individual are determined using the dental casts, radiographs and photographs as diagnostic aids.
» Bonding and stabilization wire: The Sendai SFOA recommends bonding 0.022-in brackets one week prior to the surgery. The 0.018 x 0.025-in stainless steel wires are bent passively and are inserted followed by soldering of surgical hooks, to facilitate intermaxillary fixation during the surgery.
» Model surgery: The traditional facebow records are obtained and models are simulated according to the set treatment goals as determined from the prediction tracings. The surgical splint is then fabricated to maintain the interim transit malocclusion (ITM) post-surgically. Sendai SFOA does not recommend achieving three point occlusal contact during mandibular surgery as this may result in posterior lengthening of the ramus, which has a high relapse tendency.
» Surgical splint: The surgical splint may be placed in the mandibular arch especially in cases of maxillary surgery. It consists of four ball hooks and a lingual arch.

**b. Surgical:**

Sendai SFOA recommended the modified bilateral sagittal split osteotomy combined with a T-shaped miniplate fixation for mandibular surgery.^19^ This design prevents the condylar dislocation due to a buccal step adjacent to mandibular second molar area, hence minimizing the relapse tendency. The titanium miniplates are placed at appropriate locations to facilitate the dental movements. 

**c. Post-surgical orthodontics:**

Removable Gelb-type splint is maintained for about four to six weeks after the surgery. Various dental movements in sagittal, vertical and transverse planes are achieved using SAS after the removal of splint.

### Variations in surgical protocols for SFOA

Over the period of years, various clinicians have modified the original SFOA, according to their clinical expertise, skills and convenience:

» Diagnosis: Various technologies such as CBCT, intraoral scans and combining these to form a 3D virtual model are being utilized to facilitate the diagnostic procedure. Swennen et al^20^ and Choi et al^21^ have reported that the use of 3D techniques would result in an accurate diagnostic work up, leading to an efficient surgical protocol and improved outcome.
» Computer-aided surgical simulation: Ima et al^22^ have recommended the usage of 3D models to simulate the jaw and future dental movements. These may also determine various interferences that may occur during the surgical procedure.
» Splint fabrication: The splints may be fabricated directly on the models^16^ ^,^ ^18^ or from virtual models using the CAD-CAM technology.^11^
» Orthodontics preparation: As opposed to the original protocol with no presurgical orthodontics, a minimal duration of presurgical orthodontic treatment may be indicated to avoid interferences during the surgical procedure.^10^

Yu et al^23^ and Villegas et al^24^ recommended that brackets should be placed one week before orthognathic surgery. Ko et al^25^ recommended bonding brackets one month before surgery.

The original Sendai SFOA has recommended leaving stainless steel wires in place post-surgically, while the stability of determined positions of the jaws have been achieved.^16^ ^,^ ^18^ However, Choi et al^21^ have recommended the use of flexible nickel-titanium wires immediately after the surgery. The use of nickel-titanium wires would result in immediate tooth movement, which can be an advantage due to regional acceleratory phenomenon. Liou et al^26^ have preferred not to place any archwires at the time of surgery.

» Post-surgical splint: While some advocate the use of the splint only during surgery, others have advocated its use anywhere between one to four weeks after surgery. Sugawara et al^18^ have employed a removable maxillary occlusal splint to stabilize the jaw position and masticatory function.
» Post-surgical orthodontics: The post orthodontic treatment may be initiated immediately post-surgery as proposed by Leelasinjaroen et al,^27^ while others suggested a delay of two-three weeks.^16,18^ Kim et al^17^ suggested to wait four-six weeks before commencing with the orthodontic treatment.

### Treatment planning considerations for SFOA

The occlusion cannot be used as a guide during the surgical procedure in SFOA. The following should be considered during the treatment planning phase to maximize the stability of the corrected jaw position:

» The model surgery should result in an ITM comprising of two occlusal stops in the posterior and one in the anterior region.^28^
» The surgical movement of the jaws should be greater as compared to the conventional orthognathic surgery, to allow for decompensation of teeth post-surgery.
» The molar relationship may be used as a guide for ITM.
» Extractions may be indicated for correction of crowding, inclinations and improvement of facial profile. Sharma et al^29^ suggested that extraction should be done if the angulation of the upper incisor to occlusal plane is less than 53 degrees. Moreover, distalization or angulating the maxillary segment during the surgical procedure may also be used to improve the teeth inclinations.
» The transverse discrepancy can be resolved either during surgery or post-surgery with archwires and elastics.
» In Class II division 2 cases, a short term period of minimal orthodontics to upright the incisors or to overcorrect the jaw deformity to Class III relations is indicated to provide sufficient overjet for surgical correction.^10^
» In Class III cases with moderate to severe crowding and retroclined incisors, the jaw deformity should be overcorrected to a Class II jaw relationship.
» In subjects with hypodivergent skeletal pattern, the deep bite can be corrected during surgery by bringing the anterior teeth into edge to edge bite with no contact between the posterior teeth. The posterior teeth are then extruded postsurgically to correct the bite.^26^ ^,^ ^29^
» In subjects with hyperdivergent skeletal pattern, the anterior open bite is corrected by clockwise rotation of maxilla and anticlockwise rotation of mandible to counter postsurgical relapse.^29^

### Stability of SFOA

Baek et al,^30^ Choi et al^31^ and Yang et al^32^ have found no statistically significant differences in the stability of SFOA and COS. For transverse problems, Wang et al^33^ have reported that the final treatment outcome in both SFOA and COS were similar. In the vertical plane, Liao et al^34^ have reported increased counterclockwise rotation while Kim et al^17^ found clockwise rotation of mandible in SFOA group as compared to COS group. For sagittal plane, Kim et al^35^ have found greater relapse of around 2.4 mm in SFOA as compared to 1.6 mm in COS. 

### Current trends in SFOA

The introduction of virtual treatment simulation and planning softwares utilizing 3D imaging techniques and virtual models have greatly improved the orthodontic diagnosis and predictability of the expected outcome.^12^ ^,^ ^36^ ^,^ ^37^ The rapid prototyping technology combined with SFOA has aided in virtual setup, treatment simulations and surgical splint fabrication, leading to improved treatment accuracy by eliminating the error. The 2.5 virtual model surgery (VMS) system combines information of 2D lateral and posteroanterior cephalograms and 3D virtual models.^38^ Oh et al^39^ reported improved accuracy, reduced cost and duration and complexity, as compared to the manual technique using 2.5 VMS system. Uribe et al^40^ and Ima et al^22^ utilized the 3D VMS system consisting of 3D imaging technique and virtual models for treatment of subjects with skeletal Class III and facial asymmetry. They reported improved treatment outcomes over the manual method. The 3D techniques have significantly improved the treatment outcomes, but have disadvantages of increased radiation dose, complicated computer software and high cost. 

## CONCLUSION

SFOA is an efficient and time saving technique, but it is limited to patients with minimal arch length discrepancy, normal incisor inclination and mild-moderate sagittal, vertical and transverse discrepancies. Hence, the patient selection is critical. In addition, passive wire bending is cumbersome and time consuming. The occlusion cannot be used as a guide and the entire occlusal stability is dependent upon the surgical splint. These drawbacks may be easily overcome with proper case selection, vigilant treatment planning and effective communication between the orthodontist and maxillofacial surgeon.
